# CAR-T Cell Therapy—An Overview of Targets in Gastric Cancer

**DOI:** 10.3390/jcm9061894

**Published:** 2020-06-17

**Authors:** Dominika Bębnowska, Ewelina Grywalska, Paulina Niedźwiedzka-Rystwej, Barbara Sosnowska-Pasiarska, Jolanta Smok-Kalwat, Marcin Pasiarski, Stanisław Góźdź, Jacek Roliński, Wojciech Polkowski

**Affiliations:** 1 Institute of Biology, University of Szczecin, 71-412 Szczecin, Poland; bebnowska.d@wp.pl; 2 Department of Clinical Immunology and Immunotherapy, Medical University of Lublin, 20-093 Lublin, Poland; jacek.rolinski@gmail.com; 3 Department of Oncocardiology, Holy Cross Cancer Centre, 25-734 Kielce, Poland; spbasia@gmail.com; 4 Department of Clinical Oncology, Holy Cross Cancer Centre, 25-734 Kielce, Poland; jolantasmok1@gmail.com (J.S.-K.); stanislawgozdz1@gmail.com (S.G.); 5 Department of Immunology, Faculty of Health Sciences, Jan Kochanowski University, 25-317 Kielce, Poland; marcinpasiarski@gmail.com; 6 Department of Hematology, Holy Cross Cancer Centre, 25-734 Kielce, Poland; 7 Faculty of Medicine and Health Sciences, The Jan Kochanowski University, 25-516 Kielce, Poland; 8 Department of Surgical Oncology, Medical University of Lublin, 20-080 Lublin, Poland; wojciechpolkowski@umlub.pl

**Keywords:** CAR-T, gastric cancer, cancer therapy

## Abstract

Gastric cancer (GC) is one of the most commonly diagnosed malignancies and, unfortunately, still has a high mortality rate. Recent research points to CAR-T immunotherapy as a promising treatment for this disease. Using genetically engineered T cells designed to target a previously selected antigen, researchers are able to harness the natural anti-tumor activity of T cells. For therapy to be successful, however, it is essential to choose antigens that are present on tumor cells but not on healthy cells. In this review, we present an overview of the most important targets for CAR-T therapy in the context of GC, including their biologic function and therapeutic application. A number of clinical studies point to the following as important markers in GC: human epidermal growth factor receptor 2, carcinoembryonic antigen, mucin 1, epithelial cell adhesion molecule, claudin 18.2, mesothelin, natural-killer receptor group 2 member D, and folate receptor 1. Although these markers have been met with some success, the search for new and improved targets continues. Key among these novel biomarkers are the B7H6 ligand, actin-related protein 2/3 (ARP 2/3), neuropilin-1 (NRP-1), desmocollin 2 (DSC2), anion exchanger 1 (AF1), and cancer-related antigens CA-72-4 and CA-19-9.

## 1. Introduction

Gastric cancer (GC) is one of the most commonly diagnosed malignancies in the world, with adenocarcinoma derived from gastric mucosa cells constituting over 90% of diagnosed cases. Despite the continuous improvement of diagnostic techniques and the introduction of modern treatment methods, GC still has a high mortality rate [1]. When considering men and women combined, GC is responsible for 8.2% of cancer deaths worldwide, making it the third most common cause of death from cancer [2]. Recently, however, researchers have gained a better understanding of the molecular mechanisms involved in the pathogenesis of GC, facilitating the development of new and effective therapies [3] (Table 1).

All the clinical trials are available at www.clinicaltrials.gov (accessed on May 13, 2020).

One of the most promising treatment methods seems to be CAR-T immunotherapy, which uses genetic engineering to harness the anti-tumor activity of T-lymphocytes. First, T cells are isolated from the patient’s bloodstream. Then, they are modified using viral vectors to introduce the chimeric antigen receptor (CAR), which gives them the ability to recognize tumor-associated antigens (TAA). The resulting CAR-T cells are then infused back into the patient [4].

The CAR is specific for a particular tumor antigen. When CAR-T cells recognize the antigen, they lead to T cell proliferation and cytotoxicity, as well as the transcription of genes encoding cytokines, which causes the destruction of the target cells [5]. The CAR consists of three parts: a single-chain variable fragment (scFv), a hinge with a transboundary domain, and an intracellular component. The scFv is extracellular and is the antigen recognition domain [4,6]. It consists of the amino acid sequence of the heavy (VH) and light (VL) chains of an antibody and a short peptide that is attached to the hinge region, which affects the antigen binding capacity. The intracellular domain enables the CAR to be anchored in the cell membrane and is also responsible for signal transmission to the inside of the cell [7,8]. T cell receptors (TCR) require antigen presentation through the major histocompatibility complex (MHC), and the expression of MHC is reduced in tumors. The adoptive transfer of cells connected with CAR enables the omission of MHC-mediated antigen presentation in the signaling pathway, and therefore, the level of immune response is not lowered [9]. Choosing the right target is essential for CAR-T therapy success. The antigen should only be present on tumor cells to avoid reactions directed against healthy cells. Moreover, directing the CAR-T cells against multiple antigens at the same time increases the therapeutic effect of the method [5]. It has been demonstrated that CAR-T cells can spontaneously transfer to the tumor environment and remain there for a long time at a high concentration [10]. For this reason, the use of CAR-T cell therapy appears to be an attractive approach to complement and eventually replace currently used treatment methods. It is assumed that this is a long-term therapy; therefore, it seems to be crucial in reducing mortality among patients with GC. The development of drug resistance is also problematic and common with current treatment options, which leads to the exhaustion of all possibilities for recovery. In this case, therapy based on CAR-T cells remains the only treatment option for the patient, which is why developing this method is so important [11]. A number of clinical studies point to the following as markers of great importance for the diagnostics and functioning of GC: human epidermal growth factor receptor 2 (HER2), carcinoembryonic antigen (CEA), mucin 1 (MUC1), epithelial cell adhesion molecule (EpCAM), claudin 18.2 (CLDN 18.2), mesothelin (MSLN), natural-killer receptor group 2, member D (NKG2D), and folate receptor 1 (FOLR1). However, the high toxicity generated by the CAR-T cells creates the need to search for new potential therapeutic targets. Particular attention should be paid to biomarkers that are important for GC, including the B7H6 ligand, actin-related protein 2/3 (ARP 2/3), neuropilin-1 (NRP-1), desmocollin 2 (DSC2), anion exchanger 1 (AF1), and cancer-related antigens CA-72-4 and CA-19-9. Their use may prove to be a key element in the development of a safe and effective therapy [12]. Unfortunately, the use of CAR-T therapy has many limitations. The use of autologous T cells is expensive and requires a long production process compared to the methods currently used. There are a number of problems that reduce the effectiveness of CAR-T cell therapy in solid tumors, including the heterogeneity of tumor antigens, problems associated with tumor infiltration, and problems with proliferation and stability of the T-cells inside the tumor [13,14]. The immunosuppressive tumor microenvironment (TME) is a big challenge because it significantly weakens T cell function by overexpressing inhibitory receptors, which ultimately leads to a rapid depletion of the therapeutic cells. An additional problem is the presence of immunosuppressive cells in the TME, such as tumor-associated macrophages (TAM), myeloid-derived suppressor cells (MDSC), regulatory cells (Treg), and tumor-associated neutrophils (TAN), which facilitate tumor immune escape [15]. Moreover, *Helicobacter pylori* is an oncogenic pathogen that contributes to the development of chronic gastritis, which may lead to intestinal metaplasia and consequently to the development of GC. *H. pylori* is attributed with the ability to reduce the host immune response. It can modulate the immune response by interfering with antigen presentation, inactivating T-cell proliferation, and partially supporting T-cell apoptosis through the Human Interaction Domain 2 (VacA). Thus, *H. pylori* may reduce the effectiveness of CAR-T cell therapy [16]. However, new strategies are constantly being investigated to improve the effectiveness of CAR-T cells, which is why we are optimistic about the future of this therapy in GC.

## 2. Targets for CAR-T Cell Therapy

**Figure 1:**
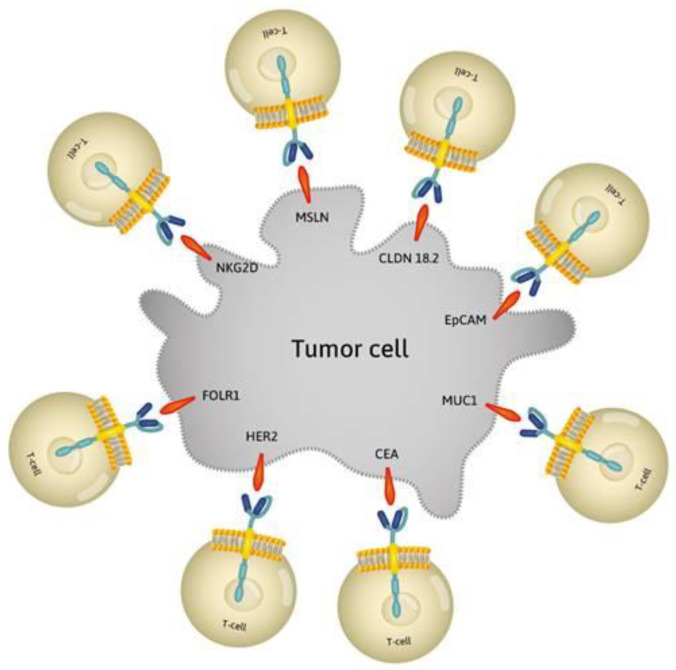
Effective targets for CAR-T immunotherapy. Modified T-cells to recognize target antigens: HER2, CEA, MUC1, EpCAM, CLDN 18.2, MSLN, NKG2D, and FOLR1 on tumor cell. HER2: human epidermal growth factor receptor 2; CEA: carcinoembryonic antigen; MUC1: mucin 1; EpCAM: epithelial cell adhesion molecule; CLDN 18.2: claudin 18.2; MSLN: mesothelin; NKG2D: natural-killer receptor group 2, member D; FOLR1: folate receptor 1.

The target antigens in CAR-T therapy should appear only on the surface of cancer cells to reduce the negative side effects that result from attacking healthy cells [17]. Studies have shown that antigens HER2, CEA, MUC1, EpCAM, CLDN 18.2, MSLN, NKG2D, and FOLR1 are effective targets for CAR-T immunotherapy [4,6] (Figure 1).

### 2.1. HER2

HER2 is a surface antigen from the epidermal growth factor receptor (EGFR) family and is encoded by the ERBB2 protooncogene, which is located on chromosome 17q21. HER2 consists of three parts: a domain containing a ligand binding site, a transmembrane domain, and a domain with tyrosine kinase activity. Receptors from the EGFR family act as signal transducers within the cell. They activate a cascade of reactions that promote cell proliferation and inhibit apoptosis, which has a significant impact on the initiation of carcinogenesis and further tumor growth [17,18]. HER2 is overexpressed in many cancers. In GC, HER2 is overexpressed in 10–20% of diagnosed cases. It is presumed that HER2 overexpression is already present in the early stages of carcinogenesis [19], and this supports GC stem cells [18]. In addition, HER2 overexpression has a negative impact on patient prognosis, increases the aggressiveness of the disease, and affects the frequency of remission and metastasis. Therefore, HER2 is an excellent therapeutic goal [19,20,21]. Trastuzumab is a commonly used therapeutic agent for HER2-positive gastric tumors, which is often used in combination with chemotherapy. About one year after starting trastuzumab treatment, resistance mechanisms that cause treatment failure appear. The development drug resistance is practically unavoidable; therefore, the implementation of new, more effective long-term therapeutic methods seems to be the highest necessity [22]. Studies have shown that the use of HER2 in CAR-T cell therapy is an effective method to treat GC, and its use reduces the need for combined therapies, as is the case with trastuzumab. HER2-directed CAR-T cells have high affinity for GC cells, even for cells with low HER2 expression. In addition, CART-HER2 cells may be an effective agent in preventing disease remission and metastasis initiation [4,18]. Other studies have reported the development of specific CART-HER2 cells, which are capable of persistent action in the cardiovascular system and of accumulating in the tumor environment, leading to its permanent inhibition [10].

### 2.2. CEA

CEA is a glycoprotein expressed on the epithelial cells of the gastrointestinal tract and lungs. It can be detected in the blood because cancer cells change their polarity [23], and for this reason it is widely used as a marker for cancer diagnosis [24]. A high level of CEA is associated with a poor prognosis and signals the possibility of metastases [25]. Research into CEA-specific CAR-T cell therapies has shown that the use of CART-CEA cells contributes to the prolongation of survival time in mice with advanced GC and the slowing down of tumor growth [4]. In addition, CEA-targeted therapy has been found to promote T cell accumulation in the tumor environment [12] without destroying healthy cells and, therefore, it constitutes an excellent therapeutic target [26]. Moreover, recent reports show that the effectiveness of CART-CEA therapy can be improved by the use of cytokines. In particular, recombinant human interleukin (IL)-12 has been shown to increase the anti-cancer activity of CAR-T cells. Therefore, a strategy using cytokines to enhance the therapeutic effect of CART-CEA should be developed [27].

### 2.3. MUC1

MUC1 is a transmembrane protein that belongs to the mucin family. Other names of MUC1 are episialin, PEM, MSA, CA15-3, KL6, DF3, MAM-6, PUM, CD227, PAS-0, and CAM 123-6 [28]. Mucins help create a protective mucosal barrier on the epithelial surface of many organs, including the stomach [29,30,31]. MUC1 is involved in intracellular signaling and can act as an adhesion ligand for stromal and endothelial cells. Thus, it affects the mobility of cells and consequently metastasis. For this reason, MUC1 is widely used to monitor the severity of metastases and tumor progression, especially in stomach cancer, with a high level indicating a poor prognosis [32,33]. Moreover, transcription of MUC1 promotes IL-11 secretion, which affects GC cell metastasis [29]. MUC-1 occurring on the surface of cancer cells shows different glycosylation patterns, which makes it an excellent target for immunotherapy [12]. Because of its involvement in oncogenesis and the high efficacy of anti-MUCH1 therapy in many cancers [34], its use as a therapeutic target in GC is very promising. CAR-T cells specific to MUC1 can effectively destroy GC tumor cells [35], but more research is needed to improve the safety of this therapy.

In September 2019, Minerva Biotechnologies opened a clinical trial to assess the use of CAR-T cells targeting MUC1* in metastatic breast cancer. Although MUC1 is expressed in solid tumors, it is cleaved and shed from the cell surface as the tumor stage progresses, which limits the effectiveness of anti-MUC1 CAR-T cells. MUC1* is an antigen that is revealed when MUC1 is cleaved. This CAR-T cell therapy uses the MNC2 antibody, which recognizes an epitope on MUC1* that is revealed when MUC1 is cleaved on tumor cells but not on healthy cells. Results of in vitro and in vivo experiments have shown CART-MUC1 cells targeting and killing breast cancer tumor cells. If tests in humans are successful, this could be a promising therapy for other solid tumors, such as GC [36].

### 2.4. EpCAM

EpCAM is a transmembrane glycoprotein also known as CD326. It is expressed in epithelial tissues, on the basolateral cell surface [37]. EpCAM plays an important role in cell signaling, differentiation, migration, and proliferation and, therefore, plays a key role in tumorigenesis and metastasis [38,39,40,41]. EpCAM is an excellent target for various therapeutic methods, including immunotherapy, because it is expressed uniformly on the whole surface of neoplastic cells [41,42]. The overexpression of EpCAM is associated with a poor prognosis in patients with multiple tumors, and its level can be used as a marker for circulating neoplastic cells (CTC) involved in metastasis [42]. EpCAM is overexpressed in >90% of gastric tumors, and consequently, its use as a therapeutic target is encouraging [38,43]. CAR-T cells directed against EpCAM were shown to be safe and effective in the treatment of solid tumors overexpressing this glycoprotein [44]. Clinical trials evaluating this therapy in the treatment of GC were completed in 2019 [43].

### 2.5. CLDN 18.2

CLDN 18.2 is the second isoform of claudine 18, located on the outer cell membrane. Under normal conditions, it is expressed in differentiated epithelial cells of the gastric mucosa, but it is also present in primary GCs [45,46]. CLDN 18.2 is expressed in 70% of primary gastric adenocarcinomas and their metastases [47], and CLDN18.2 activation is also present in pancreatic, esophageal, ovarian, and pulmonary tumors. Thus, it is considered a potential candidate for targeted GC therapy [45,46]. Jiang et al. developed CLDN 18.2-specific CAR-T cells, which persisted well in vivo, and effectively penetrated cancer tissues and did not show toxic effects in mice. More detailed studies using CAR-T cell therapy against CLDN 18.2 are ongoing as its use in the treatment of GC, as well as other CLDN 18.2-positive tumors, is promising [48]. Clinical trials to evaluate the efficacy and safety of this method in the treatment of GC in humans will end in 2021 [49].

The FDA has recently granted clearance for the use of CT041, anti-CLDN18.2 CART cells, in patients with CLDN18.2-expressing stomach, pancreatic, and gastroesophageal junction adenocarcinoma [50]. Results from a phase I clinical trial published in 2019 show a total objective response rate of 33% in a small group of patients with advanced gastric or pancreatic cancers, with no serious side effects [49]. An open-label, multicenter, Phase 1b clinical trial is planned for September 2020 in the USA to evaluate the safety and efficacy of this therapy in patients with advanced gastric or pancreatic adenocarcinoma [51].

### 2.6. MSLN

MLSN is a surface glycoprotein connected to glycophosphatidylinositol. It is a differentiation antigen with a low expression in the mesothelial cells lining the pleura, pericardium, and peritoneum [52]. MLSN may participate in cell adhesion, but its function is not fully understood [53]. MLSN is expressed in 30% of cancers, including cancers of the head, neck, esophagus, cervix, pancreas, ovary, lungs, and stomach [54,55,56]. In GC, cytoplasmic expression is more often observed than membrane expression [56]. High levels of MLSN are associated with peritoneal recurrence and can be used as a predictive factor [57]. MLSN involvement in oncogenesis may be associated with the intensification of cell proliferation and migration, promotion of neoplastic cell invasion, and metastasis by activation of PI3K, ERK, and MAPK signal pathways. MLSN also confers resistance to apoptosis induced by cytotoxic factors and has been considered a promising target in anticancer therapies [53,56,58]. Positive outcomes have been observed after using CAR-T cell therapy targeting MSLN in lung, breast, and pancreatic cancers. Moreover, MSLN-specific CAR-T cell therapy resulted in the regression of human ovarian cancer in mice, as well as an anticancer response in patients with pancreatic cancer metastases [58]. Clinical trials to assess the efficacy and safety of therapy in the treatment of malignant tumors, including GC, will be completed in 2020 [59].

### 2.7. NKG2D

The NKG2D receptor is a C-type lectin-like transmembrane glycoprotein. It occurs in human natural killer and T cells, where it acts as an activating receptor [60]. Healthy tissues do not express this glycoprotein on the cell surface. However, NKG2D ligands may be induced by various factors, such as pathogen infections, oxidative or thermal stress, genotoxic drugs, tissue damage, hyperproliferation, and malignant cell transformation. For this reason, many neoplastic cell lines and primary tumors, including GC, cause NKG2D activation. Thus, NKG2D is an excellent target for therapies such as CAR-T [61,62]. The use of modified T cells directed against NKG2D has produced good results in the elimination of lymphoma, ovarian cancer, and multiple myeloma [63]. Additionally, an advantage of this method is that the elimination of cancer cells occurs not only through the direct action of CAR-T cells, but also through the activation of host cancer immunity [63]. The immunological escape in patients with GC results mainly from the decrease in the NKG2D expression level [64]. These successes prompted researchers to develop specific CAR-T cells against NKG2D to treat GC. These cells have a strong anti-cancer action in vitro and in vivo. Additionally, the use of cisplatin increases the susceptibility of cancer cells to anti-NKG2D CAR-T cells [62]. Clinical trials evaluating CAR-T therapy targeting NKG2D in the treatment of solid tumors, including GC, will be completed in 2021 [65].

### 2.8. FOLR1

FOLR1 is a glycosylphosphatidylinositol-related protein that is also known as folic receptor alpha or folate binding protein. This protein is expressed on the surface of polarized epithelial cells, where it shows a high affinity for folic acid and enables its internalization. Although the expression of FOLR1 is low in healthy cells, it is overexpressed in many cancers, such as ovarian, breast, colorectal, kidney, lung, and other solid tumors, [66,67,68]. In cancer cells, this receptor is likely to promote proliferation and may also affect cellular signaling conducive to oncogenesis, making it a good target for targeted therapies [69,70]. Approximately one third of GC patients exhibit FOLR1 overexpression, making FOLR1-CART therapy a promising treatment. Clinical studies with CAR-T cells directed at FOLR1 conducted in ovarian cancer patients showed that this method is effective and safe. FOLR1-CAR-T cells showed high anticancer activity in preclinical studies [71]. In order to determine the effectiveness and safety of the therapy in GC patients, clinical trials are needed.

## 3. Potential Targets for CAR-T Cell Therapy

CAR-T cell therapy is associated with a number of problematic phenomena, such as the heterogeneous expression of tumor antigens, the immunosuppressive network in the tumor microenvironment, the poor migration of T cells to solid tumors, and the lack of stimulating signals necessary for CAR-T cell survival after infusion. Therefore, searching for new possible antigens is key for improving the effectiveness of therapy [6,8,9]. Factors such as B7H6, ARP2/3, NRP-1, DSC2, AE1, CA 72-4, and CA 19-9, which may have potential value in CAR-T cell therapy, should be considered [12].

### 3.1. B7H6

B7H6 is a tumor-specific ligand for the natural killer cell-activating receptor NKp30B7H6, which is absent on all healthy cells [72]. Its role is to trigger NK cell activation, and it serves as a potential recognizer in innate immunity [72]. B7H6 contributes to tumor immune evasion by engaging inhibitory receptors [72]. B7H6 expression has been observed in hematological malignancy lines, including lymphoma, leukemia, and multiple myeloma, and also in solid tumor lines, like melanoma, breast cancer, and pancreatic cancer. It has also been observed in gastrointestinal stromal tumors [73]. The absence of B7H6 in normal cells, together with its expression in tumor cells, indicates that tumor transformation upregulates B7H6. In some cases, a high level of B7H6 mRNA goes side by side with B cell lymphoma [74].

Human anti-B7H6 CAR-T cells mediated a robust cytotoxic reaction against B7H6^+^ tumors but not against B7H6^−^ tumor cells [73]. B7H6-specific CAR-T cells increased the effect of therapy in a murine lymphoma and ovarian cancer model. Gacerez and Sentman combined B7H6-specific CAR with different variants of T-bet to induce a Th1 phenotype in CD4^+^ T cells in order to increase the effectiveness of CAR-T cells [75]. Their results indicate that such a modification may impact the tumor microenvironment and trigger the immune response. 

### 3.2. ARP2/3

ARP2/3 is a seven-subunit protein complex, conserved in mammals, that plays a pivotal role in the regulation of the actin cytoskeleton in healthy cells [76]. Among other roles, ARP2/3 regulates the intracellular motility of lysosomes, endosomes, and mitochondria, and may be involved in controlling cell migration [76]. ARP2/3 is overexpressed in various tumor cells. Zheng el al. [77] assessed the expression of ARP2 and 3 in tissue samples of gastritis and gastric carcinoma and found that ARP2 was detected for 17 out of 54 gastritis and 226 out of 415 gastric carcinoma samples. ARP3 was found in 21 out of 50 cases of gastritis and 358 out of 412 gastric carcinomas. Overexpression of ARP2/3 was linked to tumor size, depth of invasion, venous invasion, and UICC staging. Therefore, ARP2/3 may have an impact on the growth, invasion, metastasis, and progression of gastric carcinomas [77].

### 3.3. NRP-1

NRP-1 is a cell surface glycoprotein that appears to be crucial for tumor angiogenesis, growth, and metastasis [78]. It is also a co-receptor regulating tumorigenesis in the vascular endothelial growth factor (VEGF)-VEGF receptor 2 and the platelet-derived growth factor (PDGF)-PDGF receptor signaling pathways [79].

A single nucleotide polymorphism (SNP) of NRP-1, the rs2065364 AA genotype, is significantly associated with improved overall survival and progression-free survival in patients with advanced GC [80]. Moreover, the number of adverse alleles for combinations of NRP-1 SNPs is an important factor for mortality and progression in advanced GC, meaning that this gene polymorphism may be a good prognostic biomarker for solid cancer [80]. Wang et al. [81] investigated the role of DNA methylation in the tumorigenesis and progression of GC and found that the hypomethylated NRP-1 strongly correlated with tumor malignant phenotypes and poor overall survival. Hang et al. also observed a correlation between the expression of NRP-1 and the expression of the microRNA molecule miR-9-5 p [82]. NRP-1 expression was reduced by the overexpression of miR-9-5 p, followed by the increased expression of the mesenchymal markers N-cadherin and vimentin and the decreased expression of the epithelial markers E-cadherin and β catenin [82]. In the course of GC, overexpression of miR-9-5 p inhibited cell proliferation, migration, and invasion [82]. The overexpression of miR-9-5 p contributed to a higher sensitivity of GC cells to cisplatin, a widely used anti-cancer drug.

### 3.4. DSC2

DSC2 is one of three glycoproteins abundant in areas subjected to stress: mainly the skin, heart, and esophagus [83]. Most GCs are positive for DSC2, and this gene is frequently upregulated in cancers with intestinal phenotype [84]. Anami et al. [84] demonstrated that 28% of GC cases were positive for this antigen, especially in GC with the intestinal mucin phenotype. Furthermore, DSC2 was correlated with CDX2 expression. These results suggest that expression of DSC2, induced by CDX2, may be a key regulator for GC with the intestinal mucin phenotype. The described results might suggest that DSC2 together with CDX2 have high potential as a therapeutic target for GC.

### 3.5. AE1 and 2

AE1 is mainly expressed on erythrocytes, and its role is to mediate the exchange of Cl-/HCO3-. AE2 is present on most tissues and regulates the intracellular pH, chloride concentration, and bicarbonate metabolism [85]. AE1 is expressed in the cytoplasm of GC cells [86] and its C-terminal 112 residues interact with the tumor suppressor p 16 [87]. Moreover, it was confirmed that p 16 also binds to AE2, and the AE1/p 16 complex induced the degradation of AE2 in GC cells. Gastrin, a major gastrointestinal hormone, could inhibit GC growth by blocking the AE1/p 16-promoted AE2 degradation [88]. Targeting AE1 with small interfering RNA (siRNA) suppresses the expression of this factor and is, thus, a potential novel approach to treating GC [89]. Also, miR-24 regulates the expression of AE1, impacting gastric carcinogenesis and erythropoiesis [90].

Expression of AE2 was also confirmed in GC cells [91] and it was shown that upregulation of AE2 in GC is dependent on early growth response 1 (EGR1) [92].

### 3.6. CA 72-4

CA 72-4 is a surface glycoprotein also known as TAG 72. It is practically never expressed in healthy tissues, but is highly expressed in cancer cells, including cells from colorectal, pancreatic, ovarian, prostate, lung, breast, and stomach cancers. The determination of the CA 72-4 level has gained high diagnostic and prognostic value in many cancers, including GC [93]. A high expression of CA 72-4 in GC patients correlates with poor prognosis, severity, and relapse [94]. The widespread occurrence of this glycoprotein in cancer makes it a promising objective for targeted therapies. For example, specific CAR-T cells directed against CA 72-4 were developed and proved to be effective in eliminating ovarian cancer cells [95]. This was also attempted in the treatment of colorectal cancer [96]. These studies should be continued, as should the method used in the treatment of other solid tumors, such as stomach cancer.

### 3.7. CA 19-9

CA19-9, also known as sialyl-Lewis, is a glycoprotein associated with Lewis antigens. It is expressed in the epithelial tissues of many organs, including the stomach. It is speculated that this antigen affects the initiation of apoptosis in activated T cells, which affects the initiation of carcinogenesis. It also plays an essential role in the intercellular adhesion of neoplastic cells, thus demonstrating a metastasis-promoting effect [94,97]. CA 19-9 is routinely used as a diagnostic marker in many cancers, including GC [98]. However, due to the low sensitivity and specificity of the tests, the effectiveness of this antigen in diagnosing the early stages of the disease remains limited [99]. An elevated serum concentration of CA 19-9 occurs in 7.3–18% of GC cases and correlates with the risk of tumor relapse and poor prognosis for patients. CA 19-19 may affect tumor growth and complications in the course of GC [97,98,100]. Due to its role in the initiation and development of stomach cancer, CA 19-9 is a great potential target for personalized therapies. CAR-T cells directed against CA 19-9 showed anticancer effects in pancreatic adenocarcinoma, which suggests that it could be a potential target for the treatment of solid tumors, such as GC. This research should be continued in the future [101].

## 4. Conclusions

According to the most recent analyses, which were performed with the use of data from the Cost–Effectiveness Analysis Registry of the Tufts Medical Center and the Institute for Clinical and Economic Review’s analysis of CAR-T therapies, CAR-T provided 5.03 (95% CI: 3.88–6.18) more incremental quality-adjusted life-years than the average pharmaceutical intervention, and 4.61 (95% CI: 1.67–7.56) more than the average nonpharmaceutical intervention, while retaining similar cost–effectiveness [102]. Baumgerdner et al. concluded that CAR-T therapy breaks a pattern of stagnant efficacy growth in pharmaceutical innovation and demonstrates significantly greater incremental effectiveness and similar cost-effectiveness to prior innovations [102]. In the context of GC, no such analyses have been carried out, but the abovementioned results suggest that cost-effectiveness might be similar.

Although CAR-T cell therapy has been successful in the treatment of hematologic diseases, it has not met the same success against solid tumors. One of the major reasons for this difference is the difficulty in finding adequate tumor-specific antigens. In this study, we have presented an overview of the most important CAR-T targets that are being investigated for GC as well as several other antigens of interest. CAR-T cell therapy is a novel treatment, especially in the context of solid tumors. More research is still needed to increase our knowledge on the interactions between T-cells and tumor cells to improve the safety and efficacy of this therapy. Nonetheless, the available evidence gives hope for GC patients.

## Figures and Tables

**Table 1:** Immunotherapies used in gastric cancer.

Immune Checkpoint Inhibitors			
Target	Drug	Description	Clinical trials
CTLA-4	Ipilimumab	IgG1κ antibody against CTLA-4	NCT01585987
	Tremeliumumab	IgG2 antibody against CTLA-4	NCT03615326
PD-1	Nivolumab	IgG4 antibody against PD-1	NCT03311334
			NCT02946671
			NCT03311334
			NCT04208958
			NCT03662659
			NCT03006705
			NCT02999295
			NCT03995017
			NCT03044613
	Pembrolizumab	IgG4 antibody against PD-1	NCT03311334
			NCT04164979
			NCT04278144
			NCT04234113
			NCT04007744
			NCT03615326
PD-L1	Avelumab	IgG1 λ antibody against PD-L1	NCT02625623
			NCT03288350
			NCT03979131
			NCT03966118
			NCT03783936
			NCT01943461
			NCT03475953
			NCT03399071
	Durvalumab (MEDI4736)	IgG1κ antibody against PD-L1	NCT03579784
			NCT04221555
			NCT03780608
			NCT02734004
			NCT03539822
			NCT02572687
CTLA-4 + PD-L1	Durvalumab + Tremeliumumab	IgG1κ antibody against PD-L1 + IgG2 antibody against CTLA-4	NCT03776487
			NCT03647969
			NCT03409848
			NCT02872116
			NCT03342417
			NCT03784040
CTLA-4 + PD-1	Ipilimumab + Nivolumab	IgG1κ antibody against CTLA-4 + IgG4 antibody against PD-1	NCT03776487
			NCT03647969
			NCT03409848 NCT02872116
			NCT03342417
			NCT03784040
Tumor antigen vaccines			
Gastrin 17	G17DT	Gastrin-17 Immunogen (G17DT induced specific and affinity antgastrin antibody (AGA)	NCT02233712
			NCT02450032
			NCT02518529
			NCT00042510
			NCT02521649
			NCT00020787
URLC10 + KOC1 + VEGFR1 + VEGFR2	VEGFR	Patient with HLA-A*2402 haplotype	NCT00681577
FOXM1+ DEPDC1 + KIF20A + URLC10 + VEGFR1	OTSGC-A24	Patients with HLA-2402 haplotype)	NCT01227772
URLC10 + VEGFR1 + VEGFR2	VEGFR	Patients with HLA-A*0201 haplotype	NCT00681252
EGFRvIII	EGFRvIII	Vaccine with sargramostim (GM-CSF) or keyhole limpet hemocyanin (KLH) as adjuvant in patients with EGFRvIII-expressing cancer	NCT00023634
HER2	TAEK-VAC-HerBy	Patients with advanced HER2-expressing cancer	NCT04246671

CTLA-4: cytotoxic T-lymphocyte antigen-4; EGFR: epidermal growth factor receptor; FOXM1: forkhead box M1; DEPDC1: DEP domain containing 1; HER2: human epidermal growth factor receptor 2; HLA: human leukocyte antigen; IgG1κ: immunoglobulin G1 kappa; IgG1λ: immunoglobulin G1 lambda; KIF10A: kinesin family member 10A; PD-1: programmed death 1 protein; PD-L1: programmed death-ligand 1; TAEK-VAC-HerBy: TAEK-VAC-HerBy vaccine; URLC10: up-regulated lung cancer 10; VEGFR: vascular endothelial growth factor receptor; Ig: immunoglobulin.
